# Radiation interception, extinction coefficient and use efficiency of wheat crop at various irrigation and nitrogen levels in a semi-arid location

**DOI:** 10.1007/s40502-018-0400-x

**Published:** 2018-09-29

**Authors:** S. Pradhan, V. K. Sehgal, K. K. Bandyopadhyay, P. Panigrahi, C. M. Parihar, S. L. Jat

**Affiliations:** 1 ICAR-Indian Institute of Water Management, Bhubaneswar, Odisha 751 023 India; 2 ICAR-Indian Agricultural Research Institute, New Delhi, Delhi 110 012 India; 3 ICAR-Indian Institute of Maize Research, New Delhi, Delhi 110 012 India

**Keywords:** Radiation interception, Radiation extinction coefficient, RUE, Wheat

## Abstract

Field experiments were conducted to study the effect of irrigation and nitrogen levels on radiation use efficiency (RUE), radiation extinction coefficient (κ) and temporal variation of leaf area index (LAI) and fraction intercepted photosynthetically active radiation (fIPAR). The LAI of wheat increased with increase in irrigation and nitrogen levels. The fIPAR also followed trend similar to LAI. The LAI and fIPAR showed logarithmic relationship with R^2^ value of 0.92 and 0.93 for the years 2013–2014 and 2014–2015, respectively. The κ value varied between 0.41 and 0.78 and was significantly affected by nitrogen levels but was not influenced by irrigation levels. The grain and above ground biomass (AGB) yields of wheat were not affected significantly by irrigation levels. However, application of 160 kg N ha^−1^ (N160) registered higher grain (12–33%) and AGB (22–25%) yeilds as compared to that with application of 40 kg N ha^−1^ (N40). Similar to AGB, the total intercepted photosynthetically active radiation (TIPAR) was not affected by irrigation levels but N160 treatment registered 9–20% higher TIPAR compared to N40 treatment. The linear relationship between TIPAR and AGB revealed that 83–86% variation in AGB yield of wheat can be explained by TIfIPAR. The RUE of wheat under three irrigations (I3) was 6 and 18% higher (*P* < 0.05) than the five (I5) and two (I2) irrigation treatments, respectively for the year 2013–2014. However, there was no significant effect of irrigation on RUE of wheat in the year 2014–2015. N160 treatment registered 5–13% higher RUE than the N40 treatment. Thus wheat may be grown with three irrigations (CRI, flowering and grain filling) and 160 kg N ha^−1^ for higher RUE without significant reduction in AGB of wheat compared to five irrigation levels in semi-arid location of Delhi region.

## Introduction

Wheat is the second most important cereal crop of India covering an area of 30 million ha with a production of 94 million tons in the year 2012–2013. Water and fertilizer (nitrogen) are the two most important inputs, which greatly contribute to wheat productivity more specifically in the arid and semi-arid tract of India where wheat is grown as a dry season crop (Pradhan et al. 2014d). Traditionally, crop responses to irrigation and nitrogen levels have been reported by many workers (Bandyopadhyay et al. 2010; Pradhan et al. 2014a, b, d; Ranjan et al. 2015) but there are very few studies evaluating the integrated effect of irrigation and nitrogen supply on the ecophysiological determinants of above ground biomass (AGB) production of wheat.

The AGB production of crop is directly related to the amount of intercepted photosynthetically active radiation (IPAR) by the crop canopy during its life cycle (Monteith 1977; Abbate et al. 1997; Sandaña et al. 2009; Pradhan et al. 2014d). The AGB per unit of total IPAR is called as radiation use efficiency (RUE) (Sinclair and Muchow 1999). The RUE of cereals is constant in non-stressful environments (Gallagher and Biscoe 1978; Sinclair and Muchow 1999). Therefore, AGB produced can be expressed as a product of the cumulative IPAR during the crop growth cycle and RUE (Sandaña et al. 2009). This approach is commonly employed in radiation use efficiency based crop growth models (Ritchie and Otter 1985; Jones et al. 1991; Keating et al. 1997; Brisson et al. 2003; Stöckle et al. 2003; Aggarwal et al. 2004) and remote sensing estimation of biomass (Casanova et al. 1998). The cumulative total IPAR of crops is mostly controlled by fraction of the incoming photosynthetically active radiation by the canopy, which is a function of green leaf area index (LAI) and the efficiency with which the green leaf area intercepts solar radiation, described by the light extinction coefficient (κ) (Plénet et al. 2000; Muurinen and Peltonen-Sainio 2006; Massignam et al. 2009; Sandaña et al. 2009). Several studies have shown that total IPAR is negatively related to both water and nitrogen deficiencies in wheat (Pradhan et al. 2014d; Dreccer et al. 2000; Salvagiotti and Miralles 2008). The κ values for wheat varies between 0.37 and 0.82 (Yunusa et al. 1993; O’Connell et al. 2004; Muurinen and Peltonen-Sainio 2006). Thomas (2013) observed that effect of irrigation was not significant on κ of wheat in a semi-arid location of India. Similar to irrigation, many authors have reported that nitrogen did not effect κ significantly (Green 1987; Muurinen and Peltonen-Sainio 2006). Though there are studies on the effect of irrigation and nitrogen on total IPAR and light extinction coefficient in isolation, studies on interactive effect of irrigation and nitrogen on these parameters are limited.

Besides species and cutivars, RUE is mostly affected by the management factors such as water and nitrogen application (Sinclair and Muchow 1999; Stöckle and Kemanian 2009; Muurinen and Peltonen-Sainio 2006). Under non-stressed conditions, the RUE values of wheat varies from 1.46 to 2.93 (Gregory et al. 1992; Yunusa et al. 1993). Water stress reduces RUE by reducing the utilization of photosynthates for growth as lower intercepted phtosynthetically active radiation occurs from reduced LAI (Wilson and Jamieson 1985; O’Connell et al. 2004). Negative responses of RUE to water stress has been presented by many workers for wheat (Han et al. 2008; Li et al. 2008; Thomas 2013). RUE is also affected by the nutrient application (Sinclair and Horie 1989; Plénet et al. 2000) and among all the nutrients, nitrogen influences RUE the most (Muurinen and Peltonen-Sainio 2006). RUE reduction under lower nitrogen application conditions is related to lower specific leaf nitrogen content and RUE increases linearly with nitrogen application till the specific leaf nitrogen stays under saturating N content (Sinclair and Muchow 1999). The negative effect of nitrogen application to RUE of wheat is well documented (Pradhan et al. 2014d; Muurinen and Peltonen-Sainio 2006). However, similar to IPAR and κ, the study on interactive effect of irrigation and nitrogen on RUE is also limited.

Successful modeling of plant growth and remote sensing estimation of biomass relies on accurate description of LAI, light extinction coefficient for IPAR and RUE. Keeping these in view, the objectives of this study were to determine the interactive effect of irrigation and nitrogen on (a) temporal variation in LAI and fraction IPAR and, (b) total IPAR, grain and AGB yield, κ and RUE of wheat in a semi-arid location of India.

## Materials and methods

### Study area and experimental details

Field experiments were conducted during dry season (winter) of 2013–2014 and 2014–2015 at the experimental farm of the Indian Agricultural Research Institute (IARI), New Delhi (77°89′E Longitude, 28°37′N Latitude and 228.7 m above mean sea level), with wheat (*Triticum aestivum* L.) as test crop. The area comes under semi-arid subtropical climatic belt. The texture of the study site was sandy loam (Typic Haplustept), low in organic carbon and available nitrogen and medium in available P and K content. The bulk density varied from 1.56 to 1.74 Mg m^−3^, saturated hydraulic conductivity from 0.49 to 1.02 cm h^−1^ and saturated water content from 0.38 to 0.42 m^3^ m^−3^ in the upper 0–1.20 m soil layer. The soil moisture content varied between 26–29% at 0.33 MPa (field capacity) and 8–11% at 1.5 MPa (permanent wilting point) in different layers of 0–1.20 m soil depth.

The experiment was laid out in a split-plot design with irrigation levels as main plot treatments and nitrogen levels as sub-plot treatments, replicated three times. The subplot size was 5 × 5 m^2^. The irrigation levels were I2: two irrigations (CRI and flowering stages), I3: three irrigations (CRI, flowering and grain filling stages) and I5: five irrigations (CRI, tillering, Jointing, flowering and grain filling stages). In each irrigation, an amount of 60 mm water was applied through surface irrigation. The irrigation amount was measured by Parshall Flume. The amount of irrigation water applied for I2, I3 and I5 were 60, 120, and 240 and 120, 120 and 240 mm for the years 2013–2014 and 2014–2015, respectively. The nitrogen levels were N40: 40 kg N ha^−1^ and N160: 160 kg N ha^−1^. The source of nitrogen fertilizer was urea. Nitrogen was applied in three splits (Basal: 50% N; CRI: 25% N and flowering stage: 25% N). All the plots received recommended basal dose of phosphorous and potassium (60 kg P_2_O_5_ ha^−1^ as single super phosphate and 60 kg K_2_O ha^−1^ as muriate of potash). Wheat crop (cv. HD 2967) was sown on 26th and 18th November in the years 2013 and 2014, respectively, by a tractor drawn seed drill (at a depth of 4–5 cm) with a row spacing of 22.5 cm and seed rate of 100 kg ha^−1^. The crop was harvested on 15th and 20th April in 2013 and 2014, respectively.

### Leaf area index (LAI)

Leaf area index was measured at regular intervals using a plant canopy analyzer (LAI-2000, LI-COR, Lincoln, NE, USA). The timing of LAI observation coincided with the timing of observation for photosynthetically active radiation (PAR).

### Canopy radiation extinction coefficient (κ)

Both incoming and outgoing photosynthetically active radiation (PAR) values were measured periodically at the top and bottom of the wheat canopy throughout the season using line quantum sensor LI-191SA (LICOR Inc., Lincoln, NE, USA). The fraction intercepted PAR (fIPAR) was calculated as (Monteith 1981):

1 $$ fIPAR = \frac{Io - I}{I} $$

where Io is incident PAR at the top of canopy and I is the transmitted PAR at the bottom of the canopy.

The canopy fIPAR and LAI were related by the relationship given below (Monsi and Saeki 1953):

2 $$ fIPAR = 1 - e^{{\left( { - \kappa \times LAI} \right)}} $$

where, κ is the canopy radiation extinction coefficient and LAI is the leaf area index. The κ was determined with least-square regression by calculating the slope of the relationship between ln(1 − fIPAR) and LAI (Robertson et al. 2001) with intercept set to zero.

### Yield

The net plot (5 m × 5 m) was harvested manually by cutting the plants close to ground after leaving the border rows. The plant samples were dried and weighed for AGB yield and expressed in kg ha^−1^. Threshing of wheat was done mechanically and the grain yield was expressed in kg ha^−1^.

### Radiation use efficiency (RUE)

Values for fIPAR for each day after sowing were interpolated between actual measurements by linear interpolation throughout the crop season (Pradhan et al. 2014c, d; Saha et al. 2015). Daily incoming solar radiation was calculated by using bright sunshine hours in the Angstrom equation (Allen et al. 1998). The daily incoming solar radiation was multiplied by a factor 0.48 (Monteith 1972) to get incoming incident PAR. Then the daily incident PAR values were multiplied by corresponding daily fIPAR values to compute daily intercepted PAR (IPAR). The daily IPAR was integrated for the whole crop season to get total IPAR (TIPAR). The RUE was calculated by dividing total AGB (g m^−2^) with the TIPAR (MJ m^−2^) for the whole crop duration (Pradhan et al. 2014c, d).

### Statistical analysis

The data were statistically analyzed using analysis of variance (ANOVA) as applicable to split-plot design (Gomez and Gomez 1984). F test was employed to see the significance of the treatment effects. The difference between the means was estimated using least significance difference and Duncan’s multiple range tests at 5% probability level. Regression analyses were performed using the data analysis tool pack of MS Excel (2007).

## Results and discussion

### Weather

Mean monthly temperature, relative humidity, solar radiation, rainfall and reference evaporation (Allen et al. 1998) are presented in Table 1. The mean monthly temperature was almost similar in both the years of study except for the month of February. The mean monthly temperature of February 2014–2015 was 3.1 °C higher than that of the year 2013–2014. It coincides with the flowering and milk stage of wheat crop growth. The wheat crop growth period of 2014–2015 (315.8 mm) received significantly higher rainfall than the year 2013–2014 (169.2 mm). More specifically, March month of 2014–2015 received 201.8 mm rainfall compared to 63.5 mm of the year 2013–2014. However, the February month of 2013–2014 received 63.5 mm rainfall in four spells whereas the year 2014–2015 did not receive any rainfall for the same period. The mean monthly relative humidity was almost similar for both the years of study except for the month of February. The February month of the year 2013–2014 registered 10% higher relative humidity compared to the year 2014–2015. It could be attributed to the higher February rainfall of the year 2013–2014 compared to the year 2014–2015. The solar radiation received for the study period of the year 2014–2015 (2617 MJ m^−2^) was almost similar to the year 2013–2014 (2639 MJ m^−2^). Similarly, the reference evapo-transpiration for the study period of 2014–2015 (523 mm) and 2013–2014 (512 mm) were almost similar. The higher reference evapo-transpiration for the month of February 2014–2015 (75 mm) compared to the year 2013–2014 (59 mm) may be attributed to the higher solar radiation received during the same period corresponding to the previous period. On the whole, wheat crop of the year 2013–2014 experienced more congenial weather compared to the wheat crop of the year 2014–2015.

**Table 1:** Weather condition during the period of study

Months	Mean temperature (°C)		Mean relative humidity (°C)		Total solar radiation (MJ m^−2^)		Total rainfall (mm)		Total reference evapotranspiration (mm)	
	2013–2014	2014–2015	2013–2014	2014–2015	2013–2014	2014–2015	2013–2014	2014–2015	2013–2014	2014–2015
Nov	18.4	19.5	70	61	368	392	0.4	0	69	77
Dec	14.7	13.7	75	76	325	333	6.8	26.4	52	53
Jan	12.7	11.9	82	83	289	290	18.6	35.8	46	45
Feb	14.5	17.6	80	70	373	394	63.5	0	59	75
Mar	20.0	20.2	69	71	564	585	63.5	201.8	114	116
Apr	26.3	26.5	57	60	698	645	16.4	51.8	172	157

### Leaf area index (LAI)

**Fig. 1:**
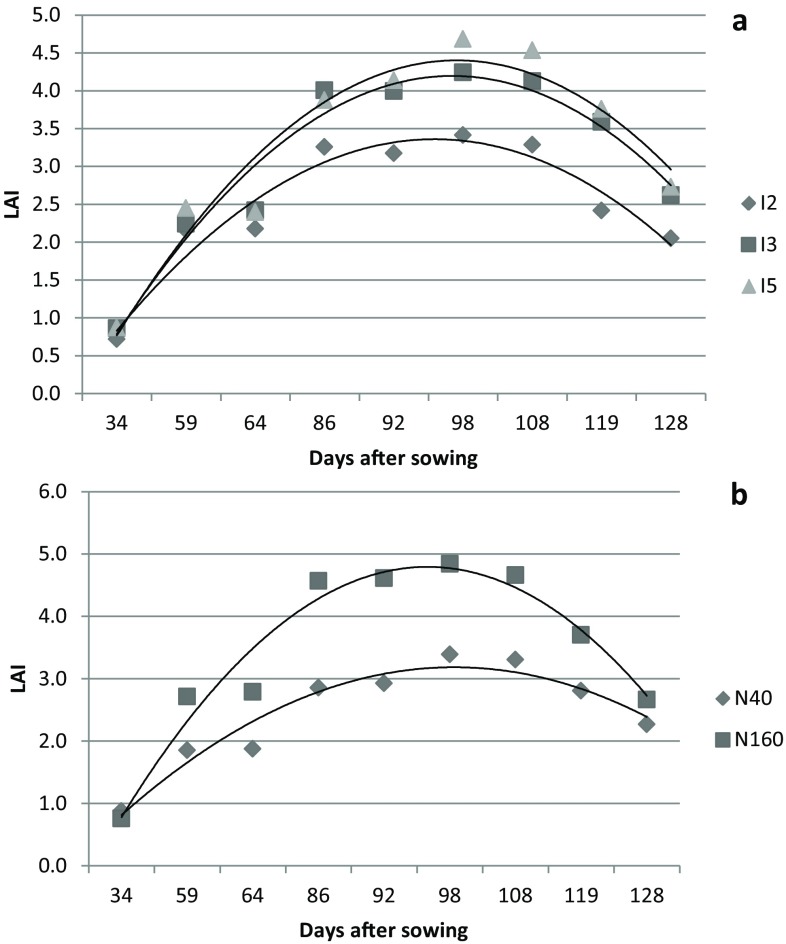
LAI variation of wheat 2013–2014 at different days after sowing (DAS) for irrigation (**a**) and nitrogen (**b**) treatments

**Fig. 2:**
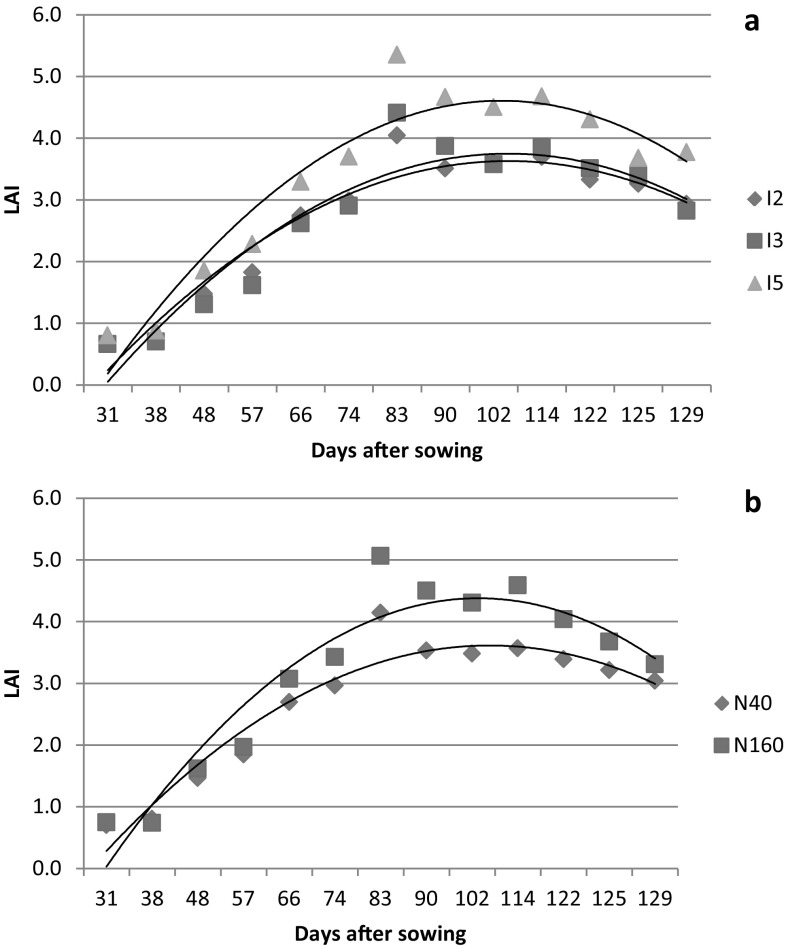
LAI variation of wheat 2014–2015 at different days after sowing (DAS) for irrigation (**a**) and nitrogen (**b**) treatments

The temporal variation in LAI of wheat crop for irrigation and nitrogen treatments of both the years of study are presented in Figs. 1 and 2. The LAI value increased steadily till 83–98 days after sowing and then declined. The increase in LAI may be attributed to foliage expansion because of development of new leaves and enlargement of existing leaves (Mandal et al. 2005). The peak value of LAI in the present experiment coincided with the booting to flowering stage of wheat. This finding is in agreement with Akram (2011) and Bassu et al. (2011) for wheat. The decrease in LAI during later part of crop growth is ascribed to leaf senescence (Thomas 2013; Mandal et al. 2005; Pradhan et al. 2013; Bandyopadhyay et al. 2010). Averaged over nitrogen treatments, the highest LAI for I5, I3 and I2 treatments were 4.69, 4.24 and 3.42 in 2013–2014 and 5.35, 4.41 and 4.05 in 2014–2015, respectively. In the year 2013–2014, the effect of irrigation levels were significant (*P* < 0.05) only on LAI measured at 128 days after sowing. However, in the year 2014–2015, irrigation effects were significant (*P* < 0.05) on LAI measured at 83, 90, 122 and 129 days after sowing. In both the years of study, the LAI of reproductive stage of wheat was highest in I5 followed by I3 and I2. Increased water stress due to differential level of irrigation application might have led to increased abscission rate and hence decreased in LAI (Akram 2011; Thomas 2013). Averaged over irrigation levels, the highest LAI for N40 and N160 treatments were 3.39 and 4.84 in 2013–2014 and 4.14 and 5.06 in 2014–2015, respectively. Nitrogen treatment significantly (*P* < 0.05) affected LAI of wheat at all stages of measurement except at 34 days after sowing for the year 2013–2014. However, in the second year, the significant effect of nitrogen levels on LAI were observed only at 83, 90, 102, 114 and 122 days after sowing. Higher LAI with increased N application could be attributed to significant increases in leaf expansion (length and breadth) resulting from cell division and cell enlargement at higher N rates. Similar results were reported by Wright (1982) and Kar and Kumar (2015) for maize, and Shafi et al. (2011) for barley. The interaction effect of irrigation and nitrogen on LAI was not significant for both the years of study.

### Fraction intercepted PAR (fIPAR)

**Fig. 3:**
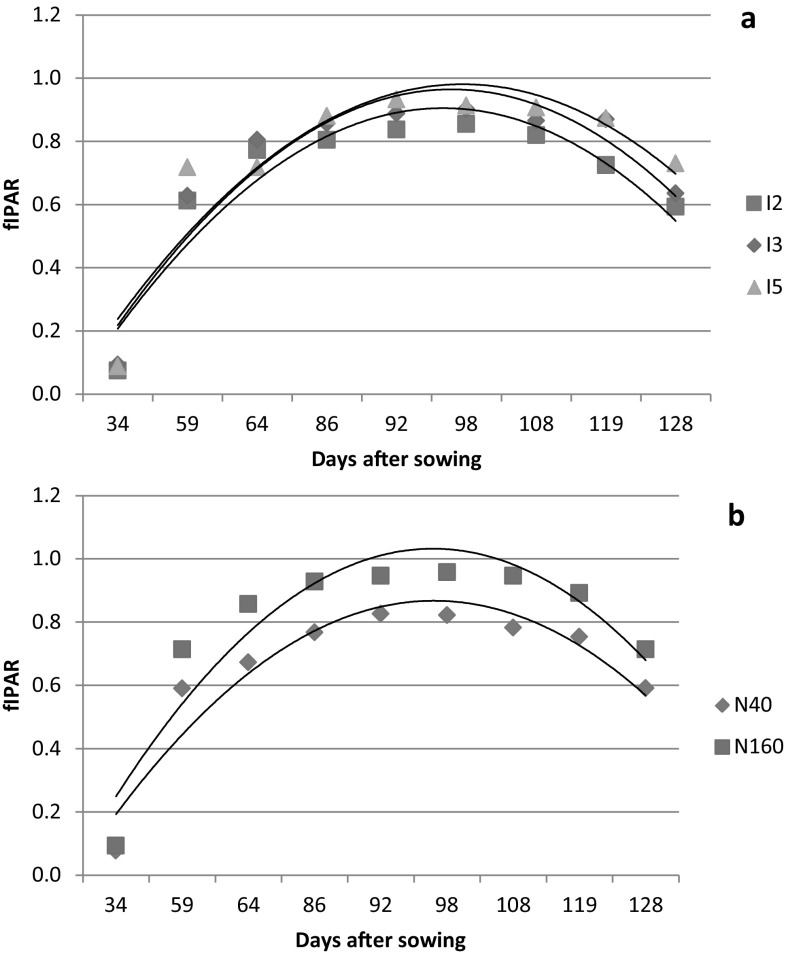
fIPAR variation of wheat 2013–2014 at different days after sowing (DAS) for irrigation (**a**) and nitrogen (**b**) treatments

**Fig. 4:**
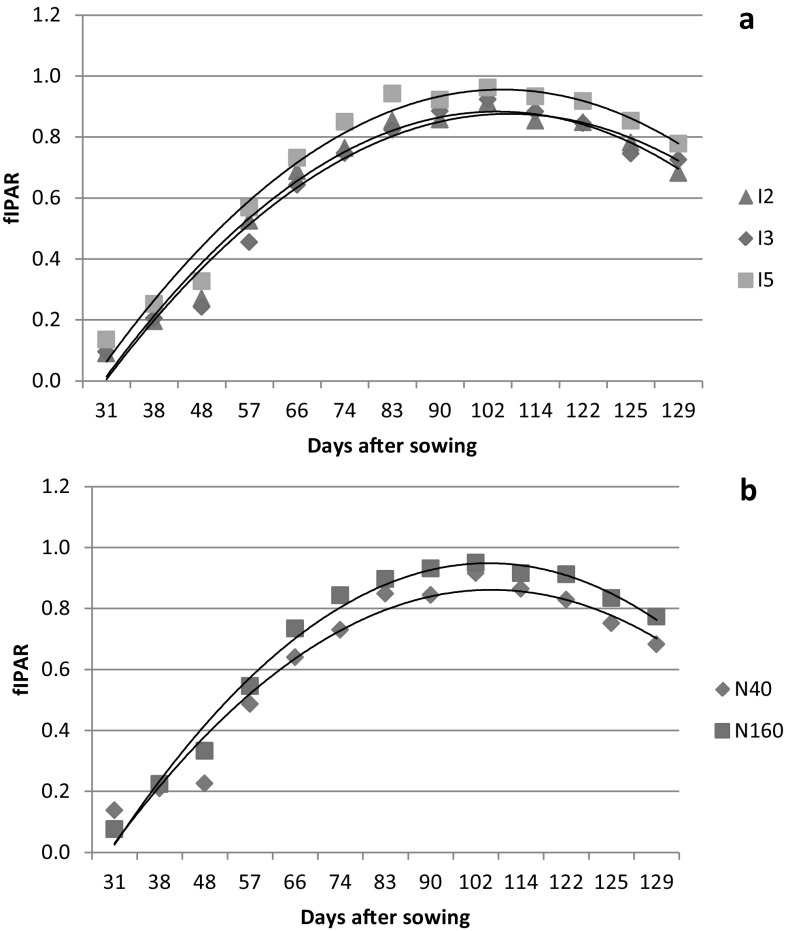
fIPAR variation of wheat 2014–2015 at different days after sowing (DAS) for irrigation (**a**) and nitrogen (**b**) treatments

**Fig. 5:**
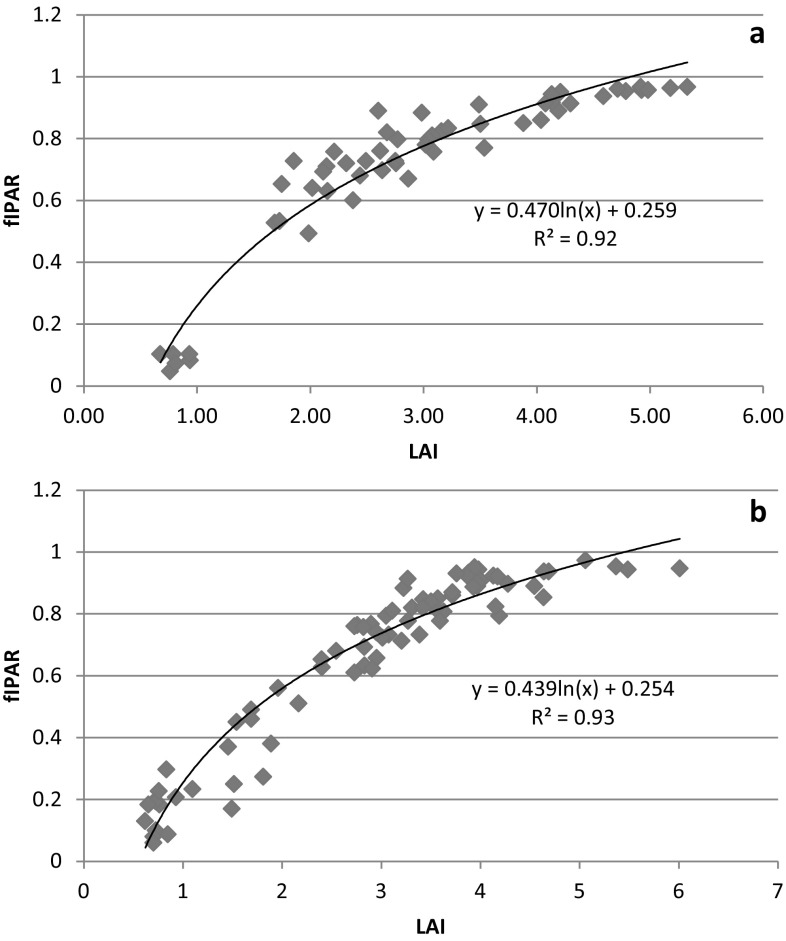
LAI and fIPAR relationship for 2013–2014 (**a**) and 2014–2015 (**b**)

The temporal variation of fIPAR of wheat for irrigation and nitrogen treatments of both the years are presented in Fig. 3 and 4. The fIPAR increased continuously till 98 DAS in 2013–2014 and 102 DAS in 2014–2015 and then decreased with progress of season. Pradhan et al. (2014c) also reported curvilinear relationship between fIPAR and days after sowing for wheat. The temporal variation of fIPAR followed the trend similar to that of LAI. Jha et al. (2012) and Serrano et al. (2000) have also observed that temporal variation in fIPAR showed the trend similar to LAI for mustard and wheat, respectively. The graphical relationship between LAI and fIPAR for both the years of study are presented in Fig. 5. The LAI and fIPAR showed logarithmic relationship with R^2^ value of 0.92 for 2013–2014 and 0.93 for 2014–2015. In both the years, fIPAR increased with increase in LAI, initially at higher rate and then at lower rate and finally flattening. This could be ascribed to the lower rate of change of fIPAR to higher rate of change of LAI after achieving the peaks of fIPAR and LAI, respectively (Thomas 2013). The irrigation levels did not affect fIPAR significantly throughout the crop growth period of 2013–2014 and 2014–2015 (Fig. 3a and 4a). Averaged over nitrogen treatments, the highest fIPAR (%) for I5, I3 and I2 treatments were 85.5, 90.2 and 91.3% in 2013–2014 and 91.3, 92.3 and 96.2% in 2014–2015, respectively. However, the nitrogen levels significantly (13–21% in 2013–2014 and 4–32% in 2014–2015) affected fIPAR at almost all stages of its measurement (Figs. 3b and 4b). Averaged over irrigation treatments, the highest fIPAR (%) for N160 and N40 treatments were 82.2 and 95.8% in 2013–2014 and 91.6 and 95.0% in 2014–2015, respectively. The lower fIPAR in N40 treatments compared to N160 treatments can be attributed to lower LAI in the former than the later. Bassu et al. (2011) has also observed lower fIPAR in durum wheat due to lower LAI. The interaction effect of irrigation and nitrogen were not significant on fIPAR for both the years of study.

### Light extinction coefficient (κ)

**Fig. 6:**
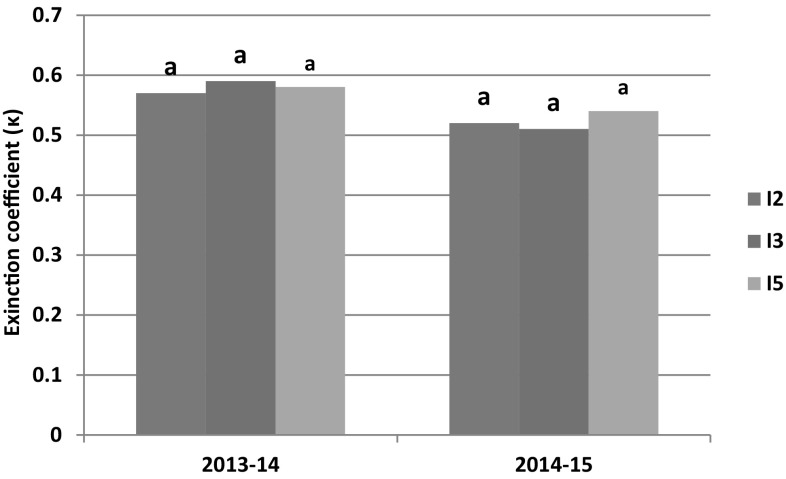
Extinction coefficient (κ) of wheat at different irrigation levels for 2013–2014 and 2014–2015. Columns marked by same letters are not significantly different at *P *< 0.05 in a year

**Fig. 7:**
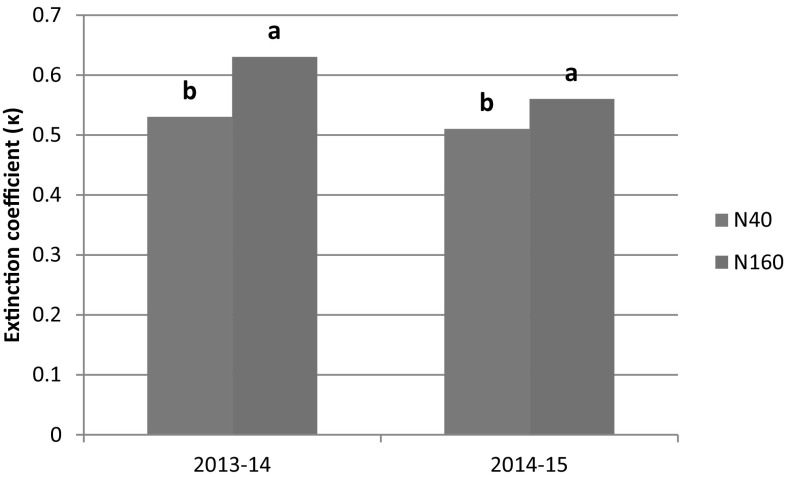
Extinction coefficient (κ) of wheat at different nitrogen levels for 2013–2014 and 2014–2015. Columns marked by same letters are not significantly different at *P *< 0.05 in a year

The canopy light extinction coefficient [the slope of ln(1 − fIPAR) and LAI relationship] was obtained for each treatment and subjected to statistical analysis and presented in Figs. 6 and 7. The κ varied between 0.51 (I2N40) to 0.65 (I3N160) in 2013–2014 and 0.47 (I2N40) to 0.58 (I2N160) in 2014–2015 (data not presented). The estimated κ values fall within the range of 0.41 and 0.78 reported for bread wheat (Yunusa et al. 1993; O’Connell et al. 2004; Muurinen and Peltonen-Sainio 2006). The irrigation levels had no significant effect on κ for both the years of study (Fig. 6a). This result is in agreement with the Thomas (2013). However, κ was significantly (*P* < 0.05) lower in N40 (16% in 2013–2014 and 9% in 2014–2015) compared to N160. It indicated that under nitrogen stress condition, the leaf becomes more erect resulting in better penetration of PAR into the canopy and hence lower fIPAR and RUE (Kiniry et al. 1989; Brekke et al. 2011; Bassu et al. 2011; Saha et al. 2015).

### Yield

The grain and above ground biomass (AGB) yields are presented in Table 2. The grain yield varied between 2750 (I2N40) and 5500 (I5N160) kg ha^−1^ with an average value of 4340 kg ha^−1^ for 2013–2014 and 3285 (I2N40) to 4001 (I5N160) kg ha^−1^ with an average value of 3732 kg ha^−1^ for 2014–2015. Similar to grain yield, the AGB varied between 8125 (I2N40) and 14,500 (I5N160) kg ha^−1^ with an average value of 11,833 kg ha^−1^ for 2013–2014 and 8625 (I2N40) to 13,500 (I5N160) kg ha^−1^ with an average value of 10,993 kg ha^−1^ for 2014–2015. The mean grain yield was 14% lower in 2014–2015 as compared to 2013–2014. However, the mean AGB yield was 7% lower in 2014–2015 as compared to 2013–2014. The decrease in yield of wheat in 2014–2015 compared to 2013–2014 can be attributed to the higher rainfall (201.8 mm) causing aeration stress during the March month of 2014–2015. Thomas (2013) has also reported decrease in yield of wheat due to aeration stress. The grain yield was not significantly (*P* < 0.05) affected by the irrigation levels for both the years of study. The N160 treatment registered 33% higher (*P* < 0.05) grain yield compared to N40 treatment in 2013–2014. However, in 2014–2015 the N160 and N40 treatments were statistically at par. Similar to grain yield, the AGB was not significantly (*P* < 0.05) affected by irrigation levels for both the years of study (Table 2). However, the AGB of wheat was significantly affected by nitrogen levels. N160 treatment registered 25% and 22% higher AGB compared to N40 treatment during the years 2013–2014 and 2014–2015, respectively. The increased yield in N160 treatment compared to N40 treatment can be attributed to increased LAI (Figs. 1 and 2), green spikes area and crop duration with greenness, which resulted increased interception of radiation (Latiri-Souki et al. 1998; Pradhan et al. 2014d). The interaction effect of irrigation and nitrogen treatments was significant on the grain yield of 2013–2014 and AGB of both the years of study. The highest grain and AGB yield was observed in I5N160 and lowest in I2N40 treatment for both the year of study. The grain yield of I5N160, I3N160 and I3N40 were statistically at par. The I3N40, I3N160, I5N40 and I5N160 treatments in 2013–2014 and I2N160, I3N160, I5N40 and I5N160 treatments in 2014–2015 were statistically at par with respect to AGB.

**Table 2:** Above ground biomass (kg ha^−1^), TIPAR (MJ m^−2^) and RUE (g MJ^−1^) of wheat for different irrigation and nitrogen levels

	Grain yield (kg ha^−1^)		Above ground biomass (kg ha^−1^)		TIPAR (MJ m^−2^)		RUE (g MJ^−1^)	
	2013–2014	2014–2015	2013–2014	2014–2015	2013–2014	2014–2015	2013–2014	2014–2015
*Effect of irrigation*								
I2	3417a	3603a	9729a	9646a	422a	474b	2.29c	2.03a
I3	4833a	3719a	12729a	10917a	469a	489b	2.70a	2.22a
I5	4771a	3875a	13042a	12417a	509a	555a	2.55b	2.23a
*Effect of nitrogen*								
N40	3722b	3520a	10500b	9903b	424b	485b	2.45a	2.03a
N160	4958a	3944a	13167a	12083a	509a	527a	2.58a	2.29a
*Effect of irrigation *×* nitrogen*								
I2 N40	2750c	3285a	8125c	8625c	367c	440c	1.97a	2.21a
I2 N160	4083bc	3920a	11333b	10667abc	477ab	508b	2.10a	2.37a
I3 N40	4375ab	3525a	11792ab	9750bc	425bc	480bc	2.04a	2.73a
I3 N160	5292ab	3913a	13667ab	12083ab	513a	499bc	2.42a	2.67a
I5 N40	4042bc	3749a	11583ab	11333abc	481ab	536ab	2.09a	2.39a
I5 N160	5500a	4001a	14500a	13500a	536a	574a	2.32a	2.71a

### Total intercepted PAR (TIPAR)

**Fig. 8:**
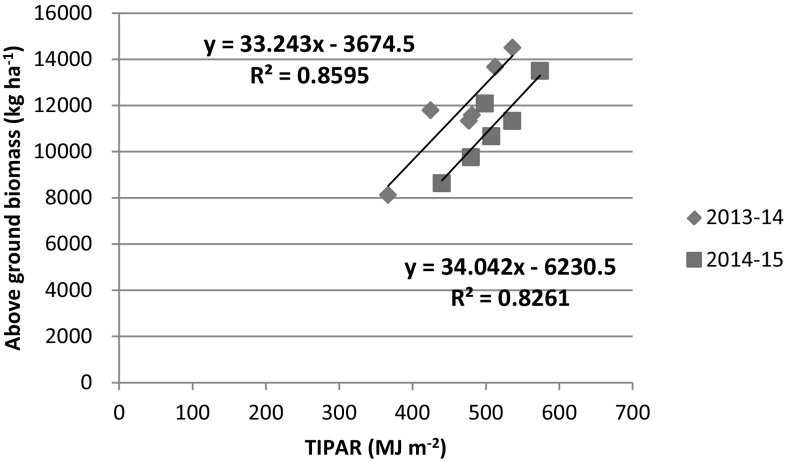
TIPAR versus above ground biomass of wheat for the year 2013–2014 and 2014–2015

The total intercepted PAR (TIPAR) is one of the most important factors of crop production (Monteith 1981). In the present experiment, TIPAR varied between 367 (I2N40) and 536 (I5N160) MJ m^−2^ with a mean value of 467 MJ m^−2^ in 2013–2014 and 440 (I2N40) to 574 (I5N160) MJ m^−2^ with a mean value of 506 MJ m^−2^ for the year 2014–2015 (Table 2). The higher (8%) TIPAR in 2014–2015 crop season compared to 2013–2014 crop season may be attributed to longer crop duration and better LAI in 2014–2015 than 2013–2014. The TIPAR was not significantly affected by irrigation levels in the first year (Table 2). However, in the second year, significantly higher TIPAR was observed in I5 (555 MJ m^−2^) compared to I3 (489 MJ m^−2^) and I2 treatments (474 MJ m^−2^), and I3 and I2 treatments were statistically at par with respect to TIPAR. The nitrogen levels significantly (*P* < 0.05) affected TIPAR for both the years of study (Table 2). N160 registered higher TIPAR than the N40 treatments by 20% and 9% for the year 2013–2014 and 2014–2015, respectively. The higher TIPAR at higher irrigation and nitrogen levels is attributed to higher LAI (Han et al. 2008; Bassu et al. 2011). The relationship between AGB and TIPAR of wheat at various irrigation and nitrogen treatments are presented in Fig. 8. These relationships are linear in nature. The TIPAR was significantly (*P* < 0.01) and positively correlated with the AGB yield of wheat (r = 0.93** for the year 2013–2014 and 0.0.91** for the year 2014–2015). The linear relationship between TIPAR and AGB depicts 86 and 83% variation in AGB yield of wheat can be explained by TIPAR for the year 2013–2014 and 2014–2015, respectively. Similar relationship has been observed in many crops (pigeon pea, chickpea, mustard, wheat, soybean, maize) by various workers (Singer et al. 2011; Pradhan et al. 2014c, d; Saha et al. 2015; Kar and Kumar (2015).

### Radiation use efficiency (RUE)

The radiation use efficiency (biomass produced per unit intercepted radiation) of wheat varied between 1.97 (I2N40) and 2.42 (I3N160) g MJ^−1^ in 2013–2014 with an average value of 2.51 g MJ^−1^ and 2.21 (I2N40) and 2.73 (I3N40) g MJ^−1^ with an average value of 2.16 g MJ^−1^ for the year 2014–2015 (Table 2). In the present experiment, our estimated RUE values were within the range of 1.20 and 2.93 g MJ^−1^ reported in literature for wheat across a range of environments (Kiniry et al. 1989; Siddique et al. 1989; Gregory et al. 1992; Gregory and Eastham 1996). In 2013–2014, the significantly highest (*P* < 0.05) RUE was observed in I3 (2.70 g MJ^−1^) followed by I5 (2.55 g MJ^−1^) and I2 (2.29 g MJ^−1^). However, in 2014–2015, I5 (2.23 g MJ^−1^), I3 (2.22 g MJ^−1^) and I2 (2.03 g MJ^−1^) irrigations levels were statistically at par (*P* < 0.05) with respect to RUE. However, even though RUE values were not significantly different among nitrogen levels (Table 2), they showed a decreasing trend (5% in 2013–2014 and 13% in 2014–2015) with decrease in N levels. It can be attributed to the lower AGB and higher root biomass in N40 which is commonly observed under stressful environments resulting lower RUE (Siddique et al. 1989; Hamblin et al. 1990; Jamieson et al. 1995). The decrease in RUE (based on AGB) among the treatments was mostly due to the variation in AGB than the variation in TIPAR. This is clear from the good correlation between the AGB of wheat with the RUE (0.84 for 2013–2014 and 0.88 for 2014–2015) than TIPAR with the RUE (0.57 for 2013–2014 and 0.61 for 2014–2015). However, the RUE showed good correlation (0.88) in 2013–2014 and poor correlation (0.47) in 2014–2015 with grain yield. The poor correlation between RUE and grain yield for 2014–2015 can be ascribed to the non-significant variation in grain yield among the treatments due to excess rainfall. These findings are in agreement with the findings of Whitfield and Smith (1989), Li et al. (2008) and Han et al. (2008) for wheat. The interaction effect of irrigation and nitrogen levels on RUE were not significant for both the years of study.

## Conclusion

It was concluded that fIPAR of wheat followed a curvilinear relationship with time similar to that of leaf area index. There was increase in fIPAR and LAI with the increase in the irrigation level up to five irrigations and with the increase in nitrogen does up to 160 kg N ha^−1^. There was no significant difference in the TIPAR among the irrigation treatments in high rainfall year but in normal rainfall years, TIPAR increased significantly up to five irrigation level. However in both the years TIPAR of wheat increased up to 160 kg N ha^−1^. There was no significant difference among the irrigation treatments with respect to extinction coefficient but it increase significantly due to increase in N dose up to 160 kg N ha^−1^. During normal rainfall year 5 irrigation with 160 kg N ha^−1^ registered highest grain and biomass yield of wheat which is at par with 3 irrigations with 160 kg N ha^−1^. However during high rainfall years though five irrigations with 160 kg N ha^−1^ registered highest biomass yield of wheat yet grain yield was not affected by irrigation and N levels. During normal rainfall years 3 irrigations registered highest RUE but in high rainfall years effect irrigation was not significant on RUE of wheat. So wheat may be grown with three irrigations at critical stages and 160 kg N ha^−1^ in the semiarid region of Delhi region without any significant yield reduction compared to five irrigations and to achieve higher radiation use efficiency under normal rainfall situation.
